# Effect of Chandler loop shear and tubing size on thrombus architecture

**DOI:** 10.1007/s10856-023-06721-7

**Published:** 2023-05-12

**Authors:** Ziqian Zeng, Tanmaye Nallan Chakravarthula, Alexei Christodoulides, Abigail Hall, Nathan J. Alves

**Affiliations:** 1 Department of Emergency Medicine, Indiana University School of Medicine, Indianapolis, IN USA; 2 Weldon School of Biomedical Engineering, Purdue University, West Lafayette, IN USA; 3 Department of Biochemistry & Molecular Biology, Indiana University School of Medicine, Indianapolis, IN USA

## Abstract

Thrombosis can lead to a wide variety of life-threatening circumstances. As current thrombolytic drug screening models often poorly predict drug profiles, leading to failure of thrombolytic therapy or clinical translation, more representative clot substrates are necessary for drug evaluation. Utilizing a Chandler loop device to form clot analogs at high shear has gained popularity in stroke societies. However, shear-dependent clot microstructure has not been fully addressed and low shear conditions are often overlooked. We herein characterized the impact of wall shear rate (126 to 951 s^−1^) on clot properties in the Chandler loop. Different revolutions (20–60) per minute and tubing sizes (3.2 to 7.9 mm) were employed to create different sized clots to mimic various thrombosis applications. Increased shear resulted in decreased RBC counts (76.9 ± 4.3% to 17.6 ± 0.9%) and increased fibrin (10 to 60%) based on clot histology. Increased fibrin sheet morphology and platelet aggregates were observed at higher shear under scanning electron microscope. These results show the significant impact of shear and tubing size on resulting clot properties and demonstrate the capability of forming a variety of reproducible in-vivo*-like* clot analogs in the Chandler loop device controlling for simple parameters to tune clot characteristics.

**Graphical Abstract:**

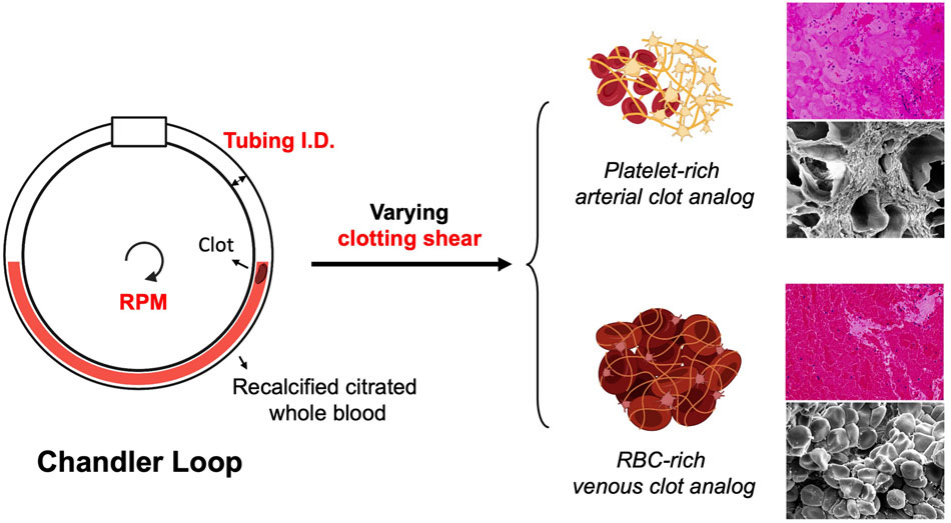

## Introduction

Varying shear conditions in blood circulation can result in the formation of thrombi with diversified clot compositions. Components of these thrombi comprise heterogenous fibrin morphology and packed cellular networks such as polyhedral red blood cells (RBCs) and platelet aggregates that often result from patient-specific physiology [1]. Formation of such structures can challenge the efficacy of a standard thrombolytic therapy leading to high risk-benefit ratios in circumstances such as deep venous thrombosis (DVT) [2]. This calls on evaluating novel drug candidates, or therapeutic regimens, using a representative drug target or system that can incorporate these properties. While animal blood clot models have enhanced the understanding of thrombosis, the cost for use of human comparable large animal models are significant and small animals, such as rodents, are not optimal for drug development as their hematological composition deviates considerably from that of humans [3]. Utilizing artificial human blood clots to study thrombolysis has attracted growing research interests in recent years [4–6]. These clot substrates formed at physiologically relevant flow conditions are believed to better capture the dynamic nature of the in-vivo thrombi and offer more accessible and representative results for use in thrombolytic drug screening.

**Fig. 1:**
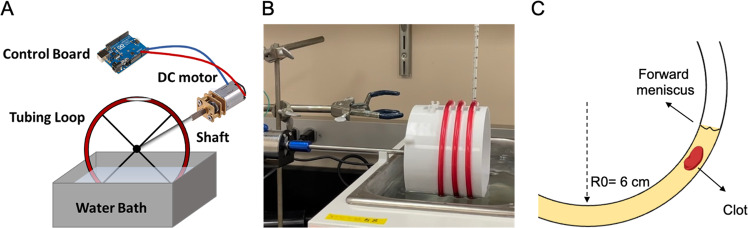
**A** Schematic representation of the Chandler loop setup. **B** A photo of the assembled Chandler loop apparatus with blood running in the device. **C** Illustration depicting clot formation within the Chandler loop at the forward meniscus

A Chandler loop apparatus is a validated tool that has previously demonstrated its capability of forming artificial clots that resemble native thrombi [7–9]. The apparatus consists of a hollow tube with ends joined to form a continuous loop, wherein the tubing is typically filled with 30–70% fluid (Fig. 1). Rotating the tubing about its center creates a torsion gradient that drives internal fluid flow against the rotational direction allowing for control over shear by adjusting tubing geometry and rotational rate. Clots formed in this device are commonly subjected to high shear yielding a structure that consists of a platelet-rich head and fibrin-rich tail, resembling the morphology of thrombi in stroke patients [7, 10]. Although wall shear rate has a known effect on clot composition within native blood, it has not yet been thoroughly characterized in the Chandler loop apparatus under low shear conditions (<500 s^−1^) where venous thrombi are commonly formed [11]. Tubing size is another important device parameter that is often overlooked despite the knowledge that lumen size-dependent flow effects, such as platelet margination and cell-free layer thickness, can contribute to clot diversity [12, 13]. Characterizing these device conditions for making artificial clots can extend the usage of the Chandler loop in preclinical drug development applications and improve the reproducibility of clots formed utilizing the device.

In this study, we explored how tubing size and wall shear rate, affect clot formation in the Chandler loop device. We compared clot weight, size, histological composition, and component distribution across nine groups comprising three tubing inner diameters (3.2, 4.8, and 7.9 mm) and three rotational rates (20, 40, and 60 RPM), whose corresponding shear rates are from 126 to 951 s^−1^ (6 groups <500 s^−1^). In addition, scanning electron microscopy (SEM) was utilized to examine clot micro-architecture and identified shear-dependent morphology of clot components. These results reveal a significant impact of tubing size and shear rates on resulting clot properties - demonstrating the capability of forming a wide variety of in-vivo-like clot analogs by modulating very simple and highly controllable clot forming parameters using the Chandler loop device. This level of control over resulting clot characteristics is not possible when utilizing purely animal thrombosis models, which often makes pharmaceutical drug optimization more complicated.

## Materials and methods

### Materials

Sodium citrate, calcium chloride, and 10% Neutral Buffered Formalin (NBF) were purchased from Sigma (St. Louis, MO). Glutaraldehyde was purchased from Fisher Scientific (Hampton, NH). Tygon® ND 100-65 biocompatible medical tubing was purchased from U.S. Plastic Corp (Lima, OH). Martius Scarlett Blue (MSB) staining kit was purchased from Avantik (Pine Brook, NJ).

### Blood sample acquisition

Animal use and care in this study were approved by the IACUC at the Indiana University School of Medicine. Ossabow swine was kept on anesthesia and mechanical ventilation during blood draw using a protocol described previously [14]. Venous blood with a 33% hematocrit was collected through the jugular vein using an 18-gauge needle into a syringe that was prefilled with 3.2% sodium citrate at a 1:9 anticoagulant to blood ratio.

### Chandler loop clot formation

A vertical Chandler loop apparatus was assembled using a DC motor (100 RPM Max) and a 3D printed spool (Creality 3D printer). A potentiometer and 12 V power supply were connected into the circuit to adjust the motor speed to provide for varying rotational rates. Clots were formed in three tubing sizes at three rotational rates each. Tubing inner diameters (R) are S = 4/32” (3.2 mm), M = 6/32” (4.8 mm), L = 10/32” (7.9 mm). Rotational rates (ω) are 20, 40, and 60 RPM. In these experiments, all tubing lumens were filled to 50% volume of blood, which are 1.5, 3.4 and 5.4 mL corresponding to the three tubing sizes. To initiate clot formation, fresh citrated blood was recalcified with 11 mM calcium chloride and transferred into the tubing. Tubing was immediately end-joined and fixed by an inner diameter to outer diameter fitted tubing segment to form a loop with a 6 cm radius of curvature (R_0_). Tubing loops were then put on the spool and rotated for 40 min, partially submerged in a 37 °C water bath. All clot formation variations were completed in a single day within 3 h of blood collection. Wall shear rates ($\dot\gamma$) were calculated by an Eq. (1) derived previously using a straight tube laminar approximation [15].

1 $$\dot \gamma = \frac{{2\pi R_0\omega }}{{15R}}$$

### Clot properties and histology

Resulting clots were gently blotted, weighed and their dimensions were measured for comparison. One clot from each group was placed in 1.5 mL of 10% NBF at RT overnight. Paraffin embedment, sectioning, and hematoxylin and eosin (H&E) staining were performed by the Indiana University School of Medicine histology core using a standard tissue-processing protocol [16]. Clot samples were cut into sections of 2 µm thickness. One slide of each group was further stained with MSB staining kit to identify platelet-rich regions of thrombi in addition to RBCs, white blood cells (WBCs), and fibrin. Images of the samples were scanned at 40X on a Zeiss Axio Scan microscope. Higher definition photos at spots of interests were taken at 40X for detailed comparison on a Leica CME microscope. Clot compositions were quantitatively analyzed using ImageJ.

### Scanning electron microscopy

SEM samples were prepared and processed as described previously [17]. Clots from each group were treated in 2.5% glutaraldehyde followed by an overnight lyophilization (FreezeZone 2.5, Labconco). Dehydrated samples were subsequently sputter coated with gold (Denton Vacuum Desktop V) for 30 s at 3*10^−4^ Torr to obtain a ~10 nm gold coating for SEM. Clot micro-structural images were taken using a field emission scanning electron microscopy (JSM-7800F, JEOL) at the Integrated Nanosystems Development Institute at Indiana University Purdue University Indianapolis. An acceleration voltage of 5 kV was applied for all samples.

### Statistical analysis

Data are described as mean ± standard deviation. Means were compared using Student’s *t*-test (equal sample size) or Welch’s *t*-test (unequal sample size) and statistical significance was reported at *P* < 0.05.

## Results

### Clot formation

Nine conditions were tested to form 3 to 4 replicates of clot analog for each condition on two identical Chandler loop apparatuses using venous blood from a single donor (Ossabow swine). In all groups, blood was able to spontaneously flow against the direction of the rotating tubing throughout the duration of the clotting process with clot formation occurring at the forward meniscus. After clotting, the remaining serum volume was measured, revealing a range of 75–86% of the starting blood volume across all groups. In addition, no apparent hemolysis was found by comparing the morphology of RBCs in the remaining liquid with those in the fresh blood (Supplementary Fig. 1).

### Clot appearance and weight

**Fig. 2:**
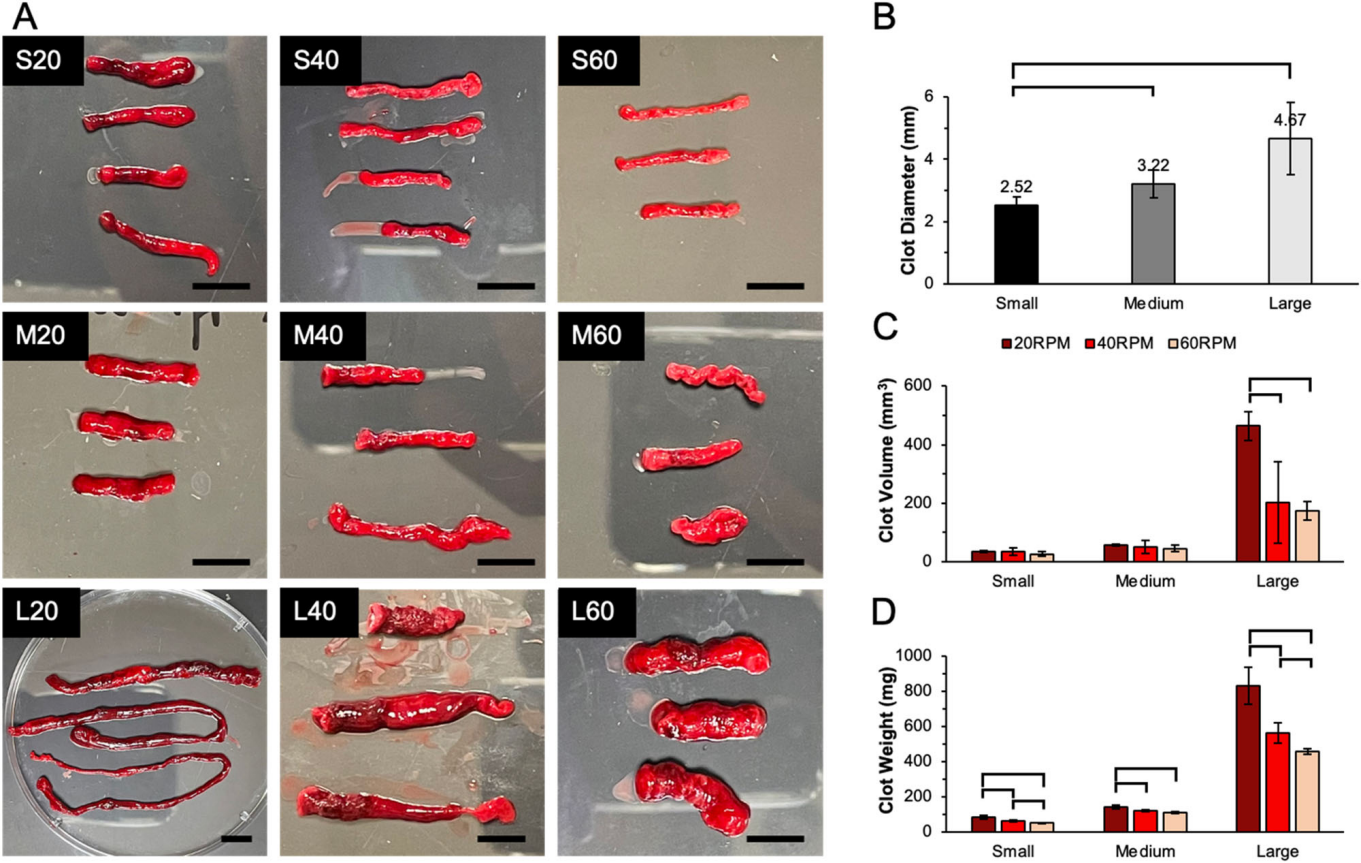
**A** Clot samples in Petri-dishes were compared across nine groups. From top to bottom: clots formed at small (S), medium (M) and large (L) tubing. From left to right: clots formed at 20, 40 and 60 RPM. Scale bars represent 20 mm. **B** Comparison of the average clot diameters formed in different diameter tubing. Diameters of clot head, body and tail were measured and averaged to report as the clot diameter for each sample. **C** Comparison of clot volume estimated through clot appearance using ImageJ. Brackets denote significant differences between groups. **D** Comparison of clot weight across groups

To examine clot appearance, clot samples were collected, gently blotted, weighed, and transferred into Petri-dishes. At higher rotational rates and smaller diameter tubing (higher shear), clots appeared to be smaller, paler, and more uniform in color. At lower rotational rates and larger diameter tubing (lower shear), clots were larger, darker, and heterogenous. The smallest clots were formed at S60 (small tube diameter at 60 RPM) while the largest clots were at L20 (large tube diameter at 20 RPM, Fig. 2). To quantitatively analyze morphological differences, clot dimensions were measured using ImageJ, clot volume and cross-sectional areas were measured utilizing calipers. The resulting clot diameter was more significantly impacted by tubing size than rotational rate (RPM). Specifically, tubing occlusion was calculated by taking a ratio of the cross-sectional area of the clot to the cross-sectional area of tubing lumen. Occlusion percentages showed significant differences (*P* < 0.05), which were 63.5 ± 13.1% in the small sized, 46.5 ± 13% in the medium sized, and 34.6 ± 15.9% in the large sized tubing. Although larger diameter tubing often led to clots with larger radii, our results indicated that clot radius is not linearly proportional to the lumen size. Clot weights were further compared, indicating a large range from 50 to 800 mg with the most massive clot formed at the slowest RPM inside the largest tubing. The variation in clot mass may also be in part due to the different blood volumes prefilled into the tubing. For example, clots formed at M60 were 1.8 times heavier (*P* < 0.05) than those formed at S40 despite these two conditions having the same calculated shear rate. Importantly, 50% lumen fill was selected in each group since using one volume may result in flow inconsistency across the wide tubing size range used in this study. However, in tubing sizes where identical blood volumes were employed, clot weight showed a significant reduction at an increased RPM (*P* < 0.05) demonstrating that high RPM, or high shear, can cause the reduction of clot weight.

### Histologic composition

Clots made in the Chandler loop were better than statically formed clots in capturing in-vivo thrombi composition and component distribution as the latter clots formed in the absence of shear provide only homogenous structures [18]. To further explore the clot compositions, one clot sample was selected from each group to be paraffin-embedded, sectioned, and stained with both H&E and MSB for subsequent analysis of clot composition as it is affected by shear and tubing size.

**Fig. 3:**
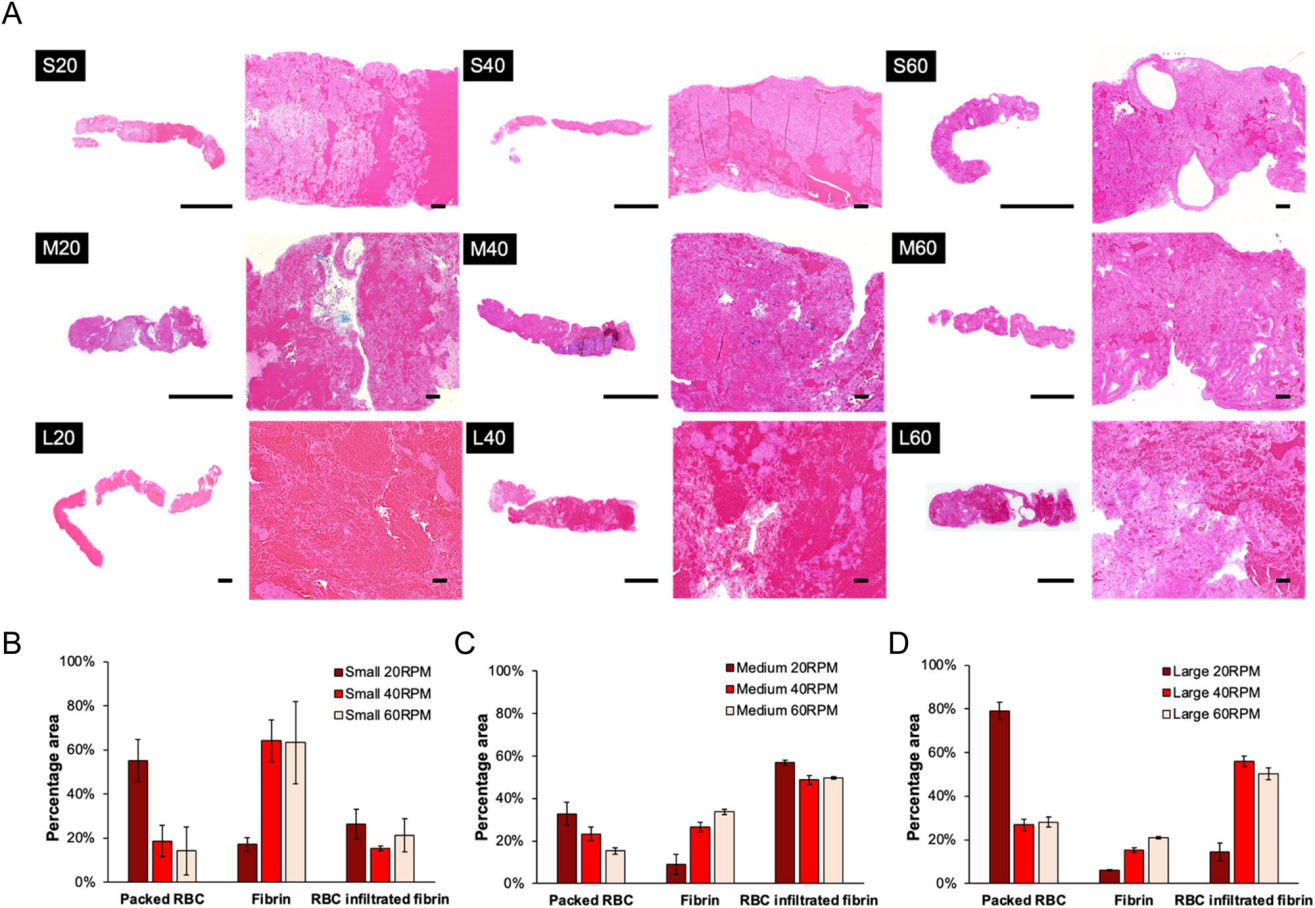
**A** Gross and 5X histologic H&E photos of representative clots formed at small, medium, and large tubing diameters at either 20, 40 or 60 RPM. H&E stains: WBCs (blue), RBCs (red), fibrin (pink). Scale bars are 5 mm in gross photos and 200 µm in 5X images. Comparing the percent area of packed RBC, fibrin-rich, and RBC infiltrated fibrin in clot samples formed at different RPMs in **B** small, **C** medium, and **D** large diameter tubing. Detailed measurements highlighting these regions of interest were included in Supplementary Fig. 2

Under H&E staining, WBCs (stained in blue), RBCs (stained in red), and fibrin (stained in pink) were differentiated (Fig. 3A). WBCs resided mostly in the fibrin (stained in pink) regions accounting for less than 3% of clot volume in all clot samples made in this study, which was consistent with the percentage found in native thrombi [19, 20]. To better compare RBCs and fibrin distribution, three related structural patterns were identified, which comprise stacked RBC regions, fibrin-rich regions, and RBC-infiltrated fibrin regions (Fig. 3B–D). Due to the employment of different blood volumes at different tubing diameters, the presence of these patterns was primarily compared across different shear rates within the same tubing size. Stacked RBC regions showed a reduction at higher RPM in all tubing groups. Fibrin-rich regions (light pink) defines a specific clot structure composed of high-density fibrin and were seen most commonly in clot heads and tails as was reported by other studies [7, 21]. In all tubing sizes, more fibrin-rich regions were observed at higher RPM. RBC infiltrated fibrin regions did not show a significant deviation in small and medium tubing groups. While in the large tubing group, L20 showed a significantly lower RBC infiltrated fibrin region than the other RPMs (*P* < 0.05), which was attributed to the relatively low fibrin incorporation at this low shear condition (126 s^−1^). In summary, an increased rotational rate results in increased fibrin-rich regions and decreased stacked RBCs.

### Shear effects on clot composition

**Fig. 4:**
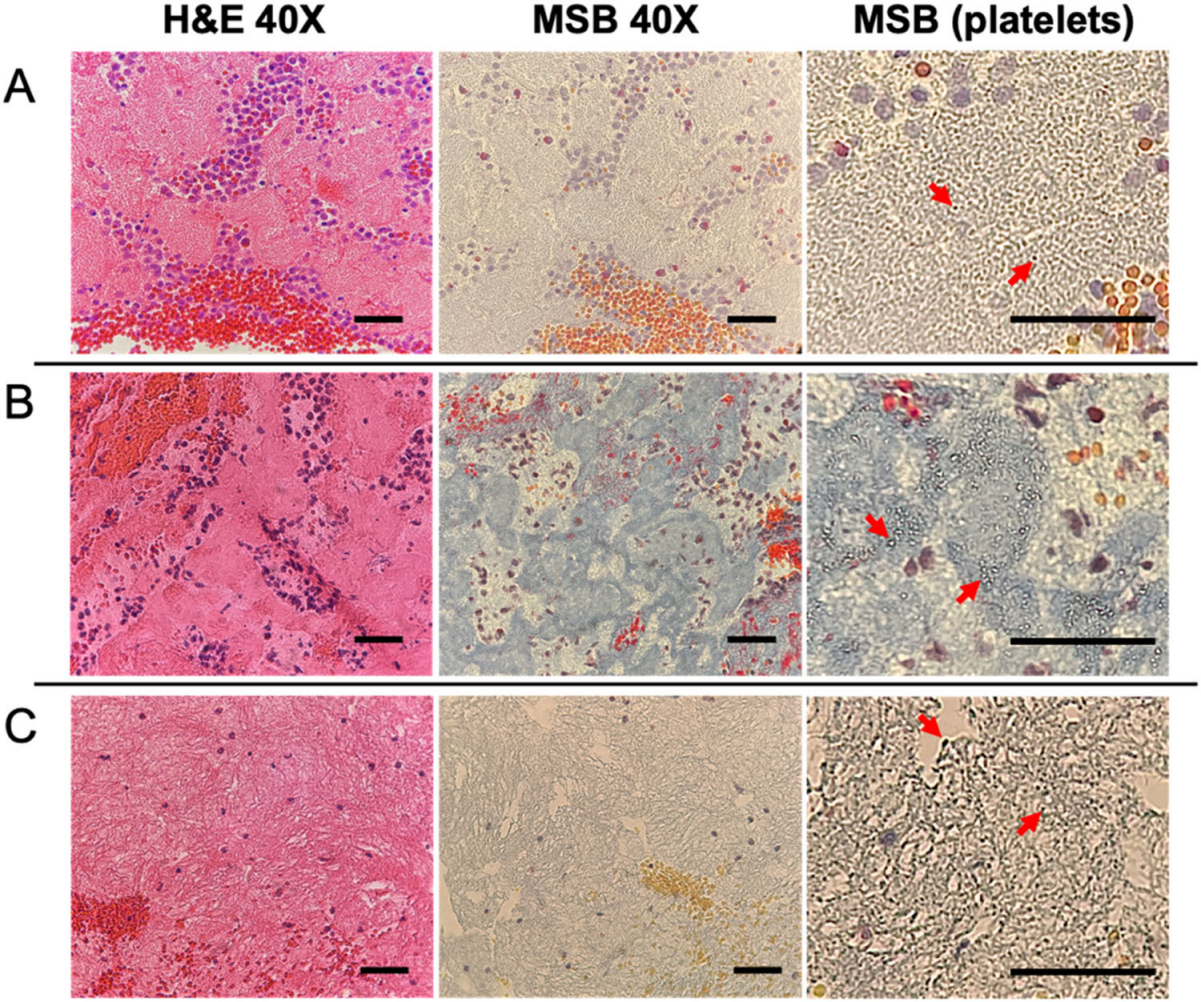
Histology photos of representative clot samples at **A** low, **B** medium, and **C** high shear rates. From left to right: photos of identical spots in the fibrin-rich region under H&E versus MSB at 40X and zoomed MSB platelet-specific regions. H&E stains: WBCs (blue), RBCs (red), fibrin (pink). MSB stains: WBCs (purple), RBCs (yellow or red), fibrin (blue or red), platelets (gray). Red arrows indicate platelets in MSB (platelets) photos. Scale bars are 5 mm for photos in the first column, 200 µm for the second column and 50 µm for the rest. Remainder clot sample photos are listed in Supplementary Fig. 3

Apart from the analysis of three defined clot structural patterns, individual components in clots were further compared based on clot forming shear rates. Therefore, data were combined and reorganized into three categories to better illustrate the effect of shear on overall clot compositions. Three shear bins were created, representing low (0–300 s^−1^; composed of L20, L40, and M20 tubing), medium (300–500 s^−1^; composed of L60, M40, and S20) and high shear (> 500 s^−1^; composed of M60, S40, and S60) (Fig. 4A–C).

Fibrin and platelets were examined using H&E and MSB staining. Fibrous fibrin network and porous fibrin were able to be differentiated using light microscopy. Porous fibrin was more frequently present at higher shear conditions. MSB staining has been regarded as the standard criterion for assessing platelet-rich regions [4]. Platelets were shown as gray granules in MSB images (Fig. 4). At low shear rates (Fig. 4A), platelets were uniformly sized and shaped, densely packed, and restricted in islands of fibrin. At high shear (Fig. 4C), platelets tended to be heterogeneous in size and mainly resided near the fibrin bundle junctions or near the edge of fibrin pores. The increased prevalence of these fibrin structures in clots formed at high shear provides for incorporation of a large number of platelets resulting in a generally fibrin and platelet-rich clot. At medium shear (Fig. 4B), the distribution of platelets comprised an equal mix of same-sized platelets found in densely packed fibrin sheets and highly contracted platelets found in the fibrous-fibrin structures. These observations could be directly attributed to platelet conformational changes in response to different shear conditions [22].

**Fig. 5:**
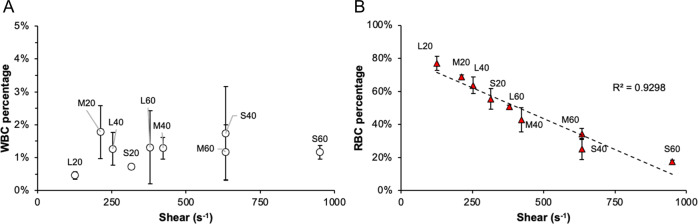
**A** WBC and **B** RBC percentage area in clots are shown over shear rates. Linear regression was plotted by RBC percentage area over increased shear rates

A quantitative analysis of WBC and RBC occupied area in these clots was also performed. Comparing to the weak trend of WBC percentage, area occupied by RBC showed an explicit negative correlation against the shear rate (R^2^ = 0.9298). Specifically, RBC percent area ranged from 17.6 ± 0.9% in S60 (highest shear) to 76.9 ± 4.3% in L20 (lowest shear, *P* < 0.05) (Fig. 5). Similar to the fact that different shear rates lead to RBC-poor or RBC-rich native thrombi formation, varying shear in the Chandler loop device also resulted in clots with different amounts of RBCs.

### Scanning electron microscopy

Native thrombi micro-structures are affected largely by thrombosis pathogenesis. A recent SEM study has reported varying compositions of micro-structures in arterial, venous, and pulmonary thrombi/emboli [1]. To further demonstrate the resemblance of clots formed in the Chandler loop device to those described clinically, SEM images of clot samples were taken to provide for a complimentary structural analysis in addition to clot histology.

**Fig. 6:**
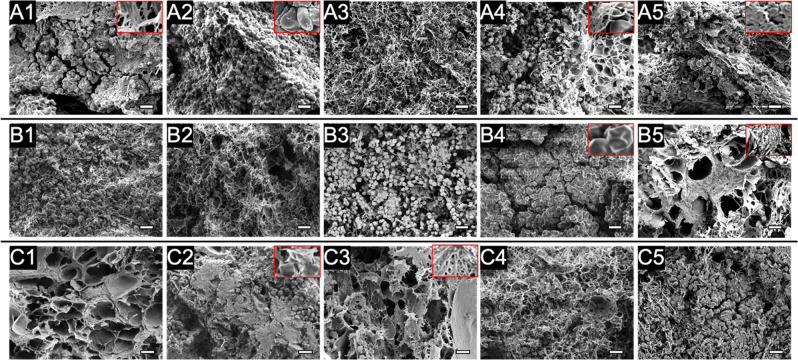
Representative SEM images of clots formed at low (**A1**–**A5**), medium (**B1**–**B5**), and high (**C1**–**C5**) shear. In each row, structures were ranked based on their high-to-low prevalence in each sample group. For example: **A1** is the most common structure in the low shear group. Images were taken at 1000X and spots of interest were shown at 2000X in selected inlaid images. Scale bars are 10 µm

Clot samples processed for SEM were sliced in the longitudinal direction to better visualize underlying clot structure [23]. This was necessary as the clot surface was covered with a thin fibrin film preventing direct visualization of internal clot morphology using intact clots. Samples were grouped into three shear levels and representative SEM images were ranked based on their presence frequency and shown accordingly (Fig. 6). Fibrin in these clots revealed a large variety of shear-dependent morphologies. Fibrin sponge (Fig. 6-C1, taken at S60 clots), sheet (Fig. 6-C3, taken at M60 clots), and bundles (Fig. 6-C4, taken at S40 clots) are the dominant motifs present at high shear compared to fibrin fibers at low (Fig. 6-A3, taken at M20 clots) and medium (Fig. 6-B2, taken at S20 clots) shear.

RBCs also showed shear-dependent morphology variations at select shear levels. Packed RBCs were observed at all shear levels under histology with these aggregating structures more clearly resolved under SEM. Importantly, RBCs were shown to be associated with minimal fibrin at low shear (Fig. 6-A1, A2, taken at L20 clots) but were largely infiltrated with fibrin fibers at medium (Fig. 6-B1, B3, taken at L20 & M40 clots) and high (Fig. 6-C5, taken at M60 clots) shear rates. Within the packed structure, RBCs were further identified as biconcave-like (Fig. 6-A2, taken at L20 clots) and polyhedral shapes (Fig. 6-B4, C5, taken at S20 and M60 clots) and quantified using a similar method published previously [1]. The predominant RBC shape was determined in a 10 × 10 µm grid in 8 to 12 SEM images that contained RBCs for each sample. Area fractions of the predominant shape to total RBCs occupied area were derived and compared across samples. Significantly more biconcave-like RBCs (28.6 ± 14.9% of total RBCs) were observed at shear rates below 500 s^−1^ compared to only 10.2 ± 1.6% at shear rates above 500 s^−1^ (*P* < 0.05). Platelets are another clot component that was identified. More platelet aggregates (Fig. 6-C1, taken at S60 clots) were resolved at high shear showing their preferrable residence in between the fibrin sheet layers. On the other hand, at low shear, very few platelets were found - most of which were surrounded by abundant RBCs (Fig. 6-A5, taken at L20 clots). In all, micro-structures and compositions of the formed clot analogs showed good resemblance to in-vivo thrombi, making them good candidates for use in thrombolytic drug testing.

## Discussion

Presented here is a study that examines a wide range of shear impacts on clotting and formation of diversified clot analogs using the Chandler loop device incorporating both tubing size and rotational rate variations in the analysis. Due to the short clot formation time and unreplenished clot components in the device, the formed clots are analogs of acute arterial and venous thrombi. Through adjusting the tubing size and rotational rate, clot size, weight, morphology, and component distribution can be precisely tuned with a high level of reproducibility, which has not been significantly addressed thus far in the literature. Formed clot samples were significantly different across experimental groups; however, properties of in-group replicates were highly consistent showing only minor variation. This was confirmed by small standard deviations shown in Fig. 2 demonstrating the Chandler loop’s high reproducibility in fabricating in-vivo-like clot analogs. Shear rates used for this study were not sufficiently high to significantly disrupt RBC membranes. Importantly, the device curvature radius was carefully selected such that the calculated wall shear rate values using the laminar approximation are also consistent with results derived using an empirical equation provided by a previously conducted numerical Chandler loop simulation [24]. Shear rates calculated by the two methods are compared in Supplementary Fig. 4.

An increased shear rate resulted in the formation of a clot with decreased RBCs, packed RBC structures and fibrin fibers, increased total fibrin, fibrin sheets and platelets based on results from both histology and SEM. The WBC percentage in clots appeared to be unaffected by varying shear conditions. RBC aggregation is a notable phenomenon at low shear, or stasis, that contributes to increased viscosity and hydrodynamic resistance in blood circulation [25]. The formation of stacked RBC structures at low shear flow conditions confirmed that the Chandler loop device can fabricate clots that resemble venous thrombi. While at higher shear, a higher fraction of rigid polyhedral RBC cellular conformation was observed that is indicative of a much tighter clot structure which was also visualized in patient thrombi samples [1, 25]. In addition, high shear promotes the formation of fibrin sheets, bundles, and sponges compared to only fibrin fibers at lower shear conditions. Although this difference in morphology has been previously addressed in in-vitro fibrin only formation studies, our results confirmed that incorporating other blood components, such as platelets and RBCs, enhanced the shear impact on fibrin morphology [26]. Tubing size was also found to significantly affect clot properties. Increasing tubing size resulted in a larger clot radius with varying proportions to tubing inner radius. Additionally, shear conditions (126–980 s^−1^) and tubing sizes (3.2–7.9 mm) employed in this study captured a wide variety of parameter combinations, which can be easily replicated in research or clinical laboratories.

This work using pig blood builds off our previous work on a swine acute pulmonary embolism model where the reinfusion of an autologous clot with a more realistic clot architecture formed by the Chandler loop can largely enhance the model fidelity [14]. Given the prevailing role of animal models for thrombosis research, forming well-controlled animal autologous clots using Chandler loop could extensively increase model and result reproducibility and should be advocated for to enhance preclinical research. Moreover, the investigation of the sole impact of Chandler loop device parameters on clot formation requires a strict control over blood biochemistry and rheology. Fresh citrated pig blood is primarily used for this purpose as obtaining the necessary volume of fresh human blood for experiments can be challenging. Pig blood functions as a good analog to human blood with similar fibrinogen levels, hematocrit, RBC size, and platelet counts [27, 28]. Pig blood has also been demonstrated to predict similar outcomes for human clot research [9]. This study would benefit from the use of human blood although similar results can be expected as the device and conditions do not need to be modified based on the source of the blood. An additional limitation of the study is that all clot formation experiments were performed at a steady flow with a fixed shear rate without replenishing blood components while native blood flow is a more complicated flow condition. Pulsatile flow can affect platelet activation that ultimately contributes to alterations in clot structures [29]. Adapting the Chandler loop device to incorporate periodic flow oscillations can likely offer a more representative clot formation condition. Likewise, replenishing blood components during clot formation in the Chandler loop provides an opportunity to investigate clot aging and the development of chronic thrombosis. However, incorporating pulsatile flow and replenishing blood components during clot formation in the Chandler loop device would significantly increase the complexity of the clot formation protocol. The versatility of the Chandler loop also makes it easy to adjust the shear stress for clot lysis applications at various shear conditions, providing a valuable tool for thrombolysis drug screening. Through combining a precisely controlled clot substrate formed in the device, researchers can effectively study the synergistic effects of thrombolytic, anticoagulant, and antiplatelet drugs. While clot analogs observed herein showed good in-vivo*-*like thrombi resemblance, the results should be carefully interpreted to guide for use in therapeutic development to model unique disease states.

## Conclusions

Ultimately, the exploration of tunable clot formation in this study promises to expand the capability of the Chandler loop device in forming diversified, reproducible, and highly controllable clot substrates. Resulting clots can have large ranges of properties that include clot weight (50–800 mg), cross-sectional diameter (2.5 to over 4.6 mm), clot RBC percentage (20–80%), fibrin-rich component (10–60%), and fibrin/cellular morphology - as demonstrated in this study. Blood component storage protocols and diversified clot formation conditions can also be employed for prolonging substrate usable life and expanding the utility to mimic pathological conditions [30–32]. These clots formed via the Chandler loop can be used directly in ex-vivo assays under static or flowing conditions for thrombolytic testing, introduced into animal models via injection as autologous clots for drug screening applications, or to study thromboresistance of medical devices such as blood pumps and artificial heart valves [33]. These diverse applications for Chandler loop formed clots capture a wide spectrum of thrombosis events that can ultimately facilitate the development of personalized treatment regimens for patients.

## Supplementary Information

Supplementary Material

## Data Availability

All supporting data are available from the corresponding author upon request.

## Abbreviations

H&E: Hematoxylin and Eosin
MSB: Martius Scarlett Blue
NBF: Neutral Buffered Formalin
RBC: Red Blood Cell
RPM: Revolutions per minute
SEM: Scanning Electron Microscopy
WBC: White Blood Cell
