# Supplementary material for: Effect of Chandler loop shear and tubing size on thrombus architecture
Source: J Mater Sci Mater Med. 2023 May 12;34(5):24. doi: 10.1007/s10856-023-06721-7 (PMC10182104; doi:10.1007/s10856-023-06721-7)
Supplement: Supplementary file 1 — Supplementary Material [file 10856_2023_6721_MOESM1_ESM.docx]

***Supplemental Material:***

**Effect of Chandler Loop Shear and Tubing Size on Thrombus Architecture**

Ziqian Zeng ^a,b^, Tanmaye Nallan Chakravarthula ^a,b^, Alexei Christodoulides ^a^, Abigail Hall ^a^ Nathan J. Alves ^a,b,c*^

^a^ Department of Emergency Medicine, Indiana University School of Medicine, Indianapolis, IN

^b^ Weldon School of Biomedical Engineering, Purdue University, West Lafayette, IN

^c^ Department of Biochemistry & Molecular Biology, Indiana University School of Medicine, Indianapolis, IN

^*^ Corresponding author:

Nathan J. Alves, PhD

Indiana University School of Medicine

635 Barnhill Dr. Rm. 2063

Indianapolis, IN 46202, United States of America

E-mail address: nalves@iu.edu (N.J. Alves)

**Table of Contents:**

**Suppl. Fig 1 RBC morphological stability**

**Suppl. Fig 2 Quantification method of structural patterns in clot samples**

**Suppl. Fig 3 Representative H&E and MSB photos for all clot samples**

**Suppl. Fig 4 Shear rates calculation**


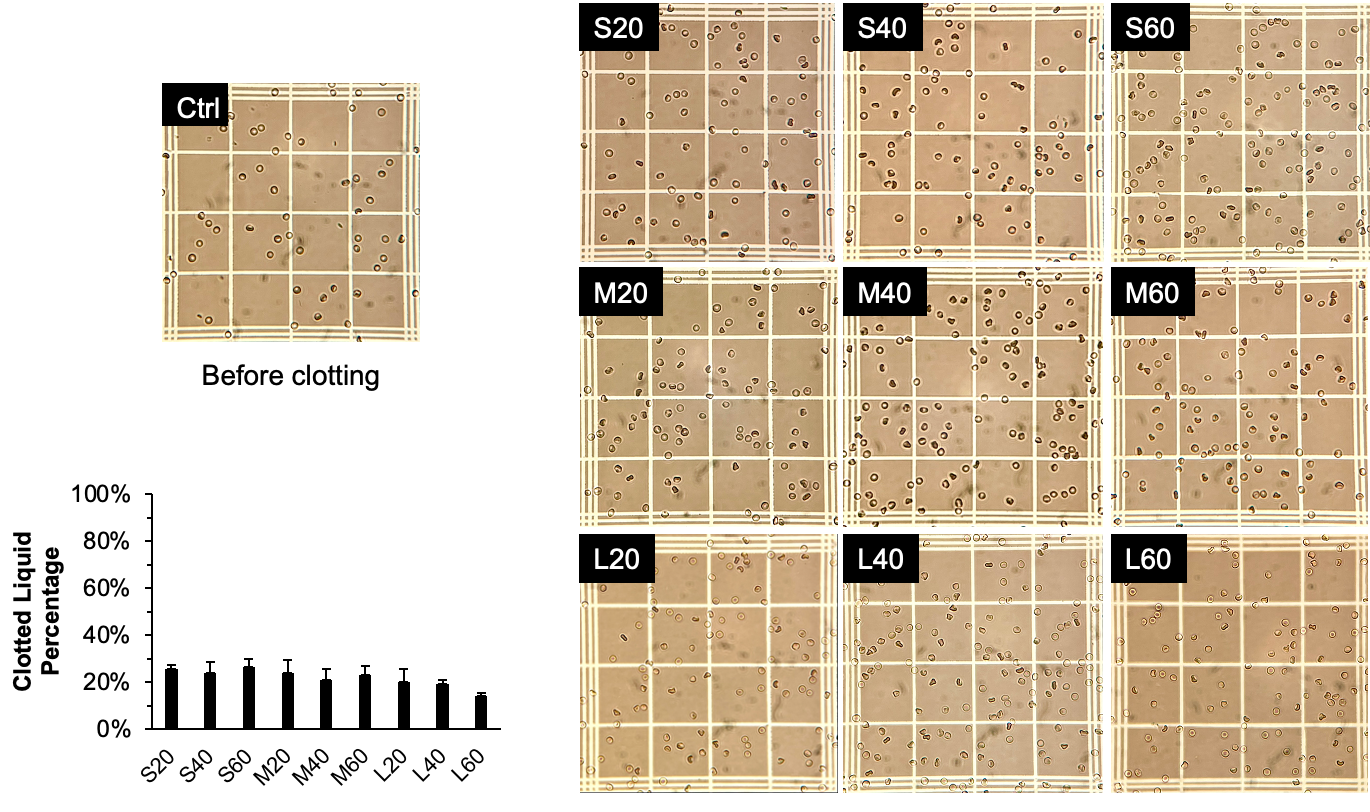


Suppl. Fig. 1 Images showing RBC morphology before and after clot formation in a hemacytometer (Bright-Line™ Hemacytometer). For RBC images after clot formation, remainder clot-free solution samples were 1:200 diluted in formalin citrate, which is an isotonic solution to preserve RBC morphology. In addition, clotted liquid volume was measured and showed an overall consistent volume ratio with respect to total solution before solution.


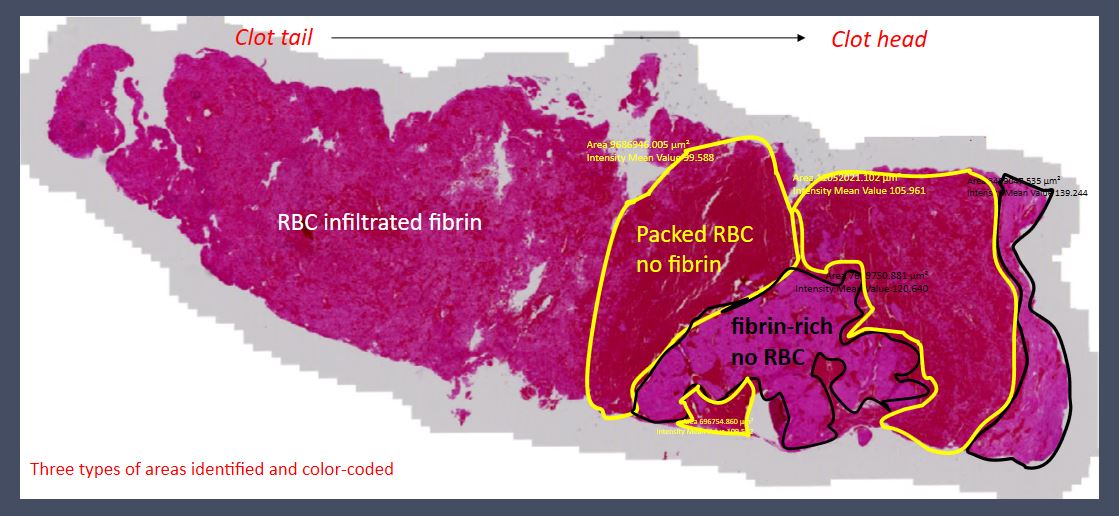


**Area quantification example (M40 sample) :**


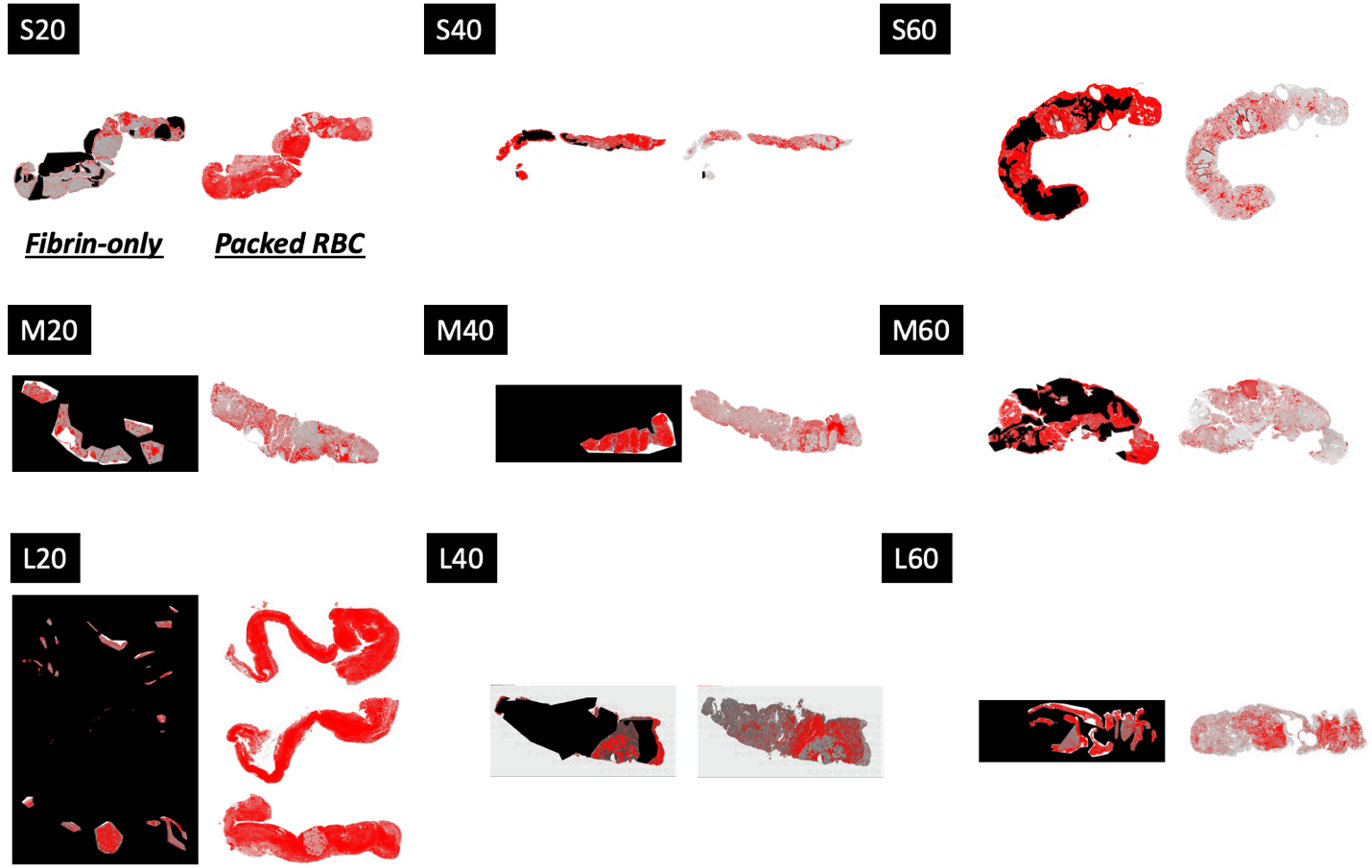


Suppl. Fig. 2 Quantification of three regions--fibrin-only, packed RBC and RBC-infiltrated fibrin in a clot sample. The demo figure shows representative regions (color coded) and corresponding area in pixel. Representative images processed using ImageJ for the area quantification of fibrin-only region (1^st^ column) and packed RBC region (2^nd^ column) in clots formed at nine different conditions (S, M, L tubing sizes and 20, 40 60 RPMs). RBC infiltrated fibrin areas were indirectly measured by subtracting fibrin-only, packed RBC and WBC areas (not shown in this figure) from total clot areas.


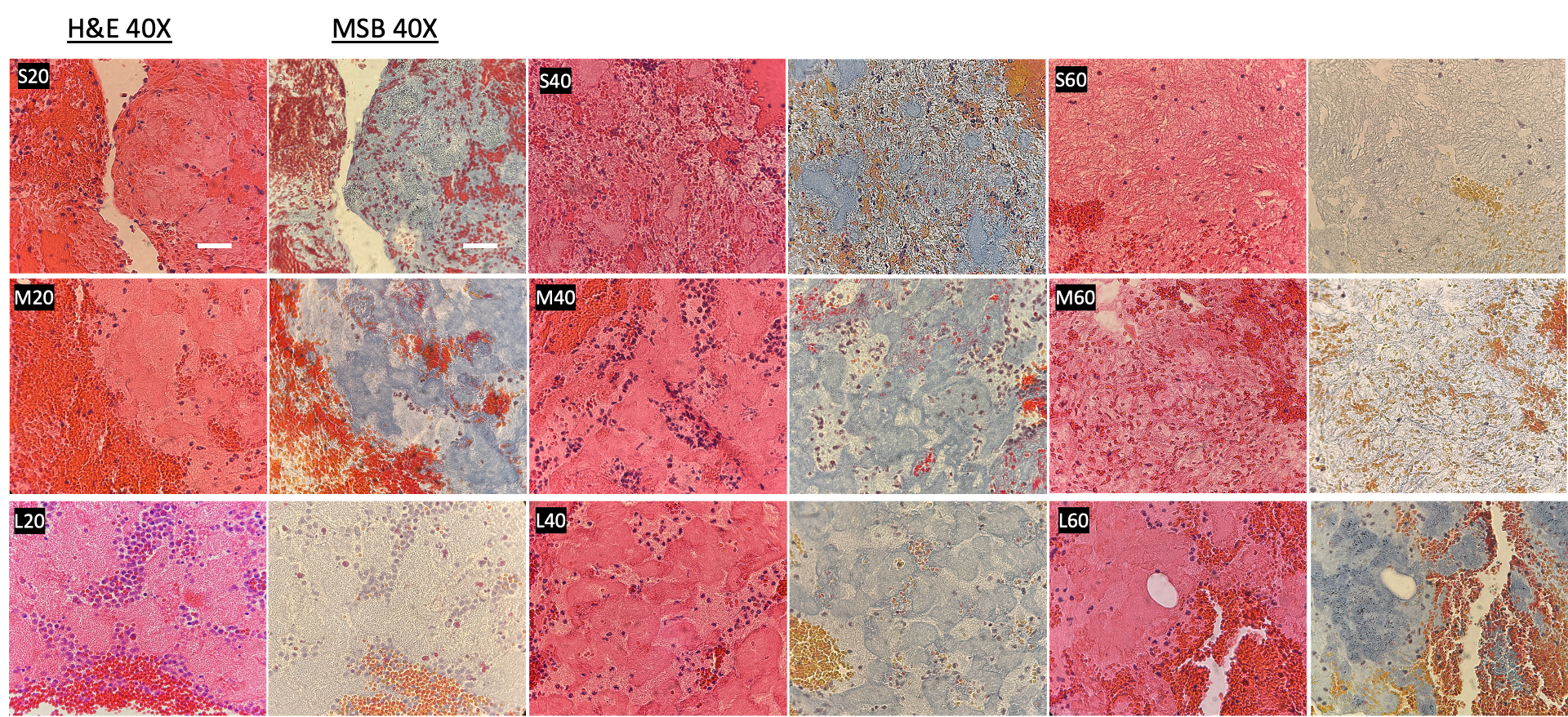


Suppl. Fig. 3 Histology photos of representative stained clot samples using H&E and MSB at 40X. H&E stains: WBCs (blue), RBCs (red), fibrin (pink). MSB stains: WBCs (purple), RBCs (yellow or red), fibrin (blue or red), platelets (gray). Scale bars are 50 µm.


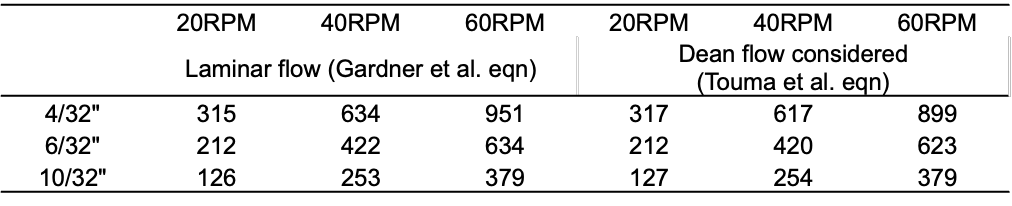

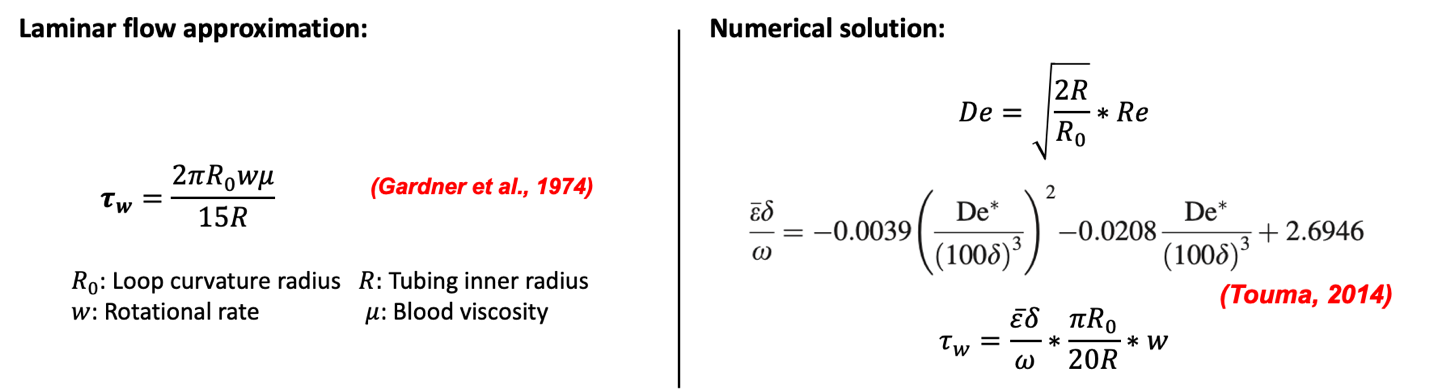


Suppl. Fig. 4 Shear rates calculation at nine different Chandler loop conditions (20, 40 and 60 RPMs, and small, medium and large tubing inner diameters) using an equation derived from laminar flow assumption by Gardner et al. (J. Lab. Clin. Med., 1974) vs an empirical equation derived by computational simulating Chandler loop published by Touma et al. (J. Biomech. Eng., 2014).
